# Copper mediated controlled radical copolymerization of styrene and 2-ethylhexyl acrylate and determination of their reactivity ratios

**DOI:** 10.3389/fchem.2014.00091

**Published:** 2014-10-17

**Authors:** Bishnu P. Koiry, Nikhil K. Singha

**Affiliations:** Rubber Technology Centre, Indian Institute of Technology KharagpurKharagpur, India

**Keywords:** controlled radical polymerization, ATRP, polystyrene, poly(2-ethylhexyl acrylate), copolymers, reactivity ratio, thermal properties

## Abstract

Copolymerization is an important synthetic tool to prepare polymers with desirable combination of properties which are difficult to achieve from the different homopolymers concerned. This investigation reports the copolymerization of 2-ethylhexyl acrylate (EHA) and styrene using copper bromide (CuBr) as catalyst in combination with N,N,N′,N″,N″- pentamethyldiethylenetriamine (PMDETA) as ligand and 1-phenylethyl bromide (PEBr) as initiator. Linear kinetic plot and linear increase in molecular weights vs. conversion indicate that copolymerization reactions were controlled. The copolymer composition was calculated using ^1^H NMR studies. The reactivity ratio of styrene and EHA (r_1_ and r_2_) were determined using the Finemann–Ross (FR), inverted Finemann–Ross (IFR), and Kelen–Tudos (KT) methods. Thermal properties of the copolymers were also studied by using TGA and DSC analysis.

## Introduction

The homopolymer and copolymers of 2-ethylhexyl acrylate (EHA) have very good film formation characteristics and have very good low temperature flexibility, because of the presence of branched and longer alkyl pendant group in the EHA. They have also low volume shrinkage. So the copolymers of EHA are widely used in paints, coating and adhesive applications (Skeist, 1977; Plessis et al., 2001; Webster and Crain, 2002). Copolymerization is an important synthetic tool which can control the thermal and mechanical properties of the polymers (Kavousian et al., 2004). In the copolymers of styrene and EHA, the properties of the copolymers can be monitored by controlled incorporation of the respective comonomers styrene and EHA. Polystyrene possess high glass transition temperature, *T*_g_ (~100°C) where as poly(2-ethylhexyl acrylate) (PEHA) has low *T*_g_ of −60°C. Copolymers of styrene and EHA are important components for hard coating (Plessis et al., 2001) and also are used as blend compatibilizers (Haldankar, 2001). Conventional free radical polymerization (FRP) leads to uncontrolled molecular weight and broad dispersity (***Đ***). There is also gel formation tendency, because of the several side reactions during FRP. This makes them difficult to apply for paints and coating material application owing to high viscosity (Solomon and Moad, 1995; Odian, 2004). Since 1990s there have been spectacular advances in the field of controlled radical polymerization (CRP). There were several CRP techniques namely, atom transfer radical polymerization (ATRP) (Kamigaito et al., 2001; Matyjaszewski and Jia, 2001; Kavitha and Singha, 2008), nitroxide-mediated polymerization (NMP) (Harth et al., 2001; Hawker et al., 2001), and reversible addition–fragmentation chain transfer (RAFT) (Chiefari et al., 1998; Moad et al., 2000; Moad, 2006; Barner-Kowollik, 2008). Among the different CRP techniques, ATRP is applicable to polymerize wide range of monomers and can be carried out at wide range of temperature (−20 to 200°C) (Haloi et al., 2009). ATRP has been successfully applied to synthesize a wide range of polymers with varied molecular weights, different architectures, functionalities etc. Transition metal catalyzed CRP known as ATRP is an important CRP method (Matyjaszewski, 2000) which is carried out in presence of an active alkyl halide using a transition metal halide as catalyst in combination with a suitable ligand (Matyjaszewski and Jia, 2001). A wide variety of copolymers can be prepared via ATRP with controlled molecular weight, functionality, and low dispersity. There are several reports on the copolymerization of different acrylate monomers using ATRP and determination of their reactivity ratios. The reactivity ratios are important parameters for a set of monomer. The reactivity ratios of monomers predict the copolymer composition as well as the sequence distribution of the comonomers. It also predicts the properties of the copolymer. ATRP provides random copolymer with similar chain compositions which is very much different from FRP (Matyjaszewski, 2002). During the polymerization reaction, the polymer chains grow simultaneously and thus all the polymer chains have same composition. However, in FRP macromolecular chains start growing at different times during the polymerization and the monomer composition continuously changes. As a result, in FRP the different chains will have different compositions in the end (Solomon and Moad, 1995). The reactivity ratios of co-monomers in a CRP are somewhat different from the same in FRP. This is because in CRP processes there is intermittent activation-deactivation of the active species which results in different rates of consumption of comonomers (Matyjaszewski, 2002; Braunecker and Matyjaszewski, 2007). For example, Mignard et al. reported the reactivity ratios of the copolymerization of styrene and butyl acrylate (BA) via NMP at 120°C in solution. They reported the reactivity ratios for styrene and BA within the range of 0.60–1.2 (r_styrene_) and 0.16–0.29 (r_BA_) respectively (Mignard et al., 2004). Arehart and Matyjaszewski reported the reactivity ratios of styrene and BA prepared via ATRP at 110°C in solution. They reported the reactivity ratios for styrene and BA as 0.68 < r_styrene_ < 0.82 and 0.22 < r_BA_ < 0.26 respectively (Arehart and Matyjaszewski, 1999). Chambard et al. reported the copolymerization of styrene and BA prepared in bulk via FRP. The reactivity ratios of styrene and BA prepared via FRP at 90°C were reported to be 0.95 and 0.20 respectively (Chambard et al., 1999). Ziegler and Matyjaszewski reported the variation in reactivity ratios of MMA and BA with the change in ligand from 4,4′-di(5-nonyl)-2,2′-bipyridine (dNbpy) (r_MMA_ = 2.52, r_BA_ = 0.26) to N,N,N′,N″,N″-pentamethyldiethylenetriamine (PMDETA) (r_MMA_ = 3.15, and r_BA_ = 0.37) (Ziegler and Matyjaszewski, 2001). Lessard et al. reported the reactivity ratios of styrene and *tert*-butyl acrylate(*t*-BA) prepared via NMP at 115°C in bulk as *r*_t-BA_ = 0.09–0.12 and r_Styrene_ = 0.40–0.49 (Lessard et al., 2007). We reported the ATRP of furfuryl methylacrylate and methyl methacrylate (Kavitha and Singha, 2007), 2-ethylhexyl acrylate and glycidyl methacrylate (Haloi et al., 2009). However, there is no report on the ATRP of styrene and EHA and to determine their reactivity ratios. The objective of this investigation is to study the copolymerization of styrene and EHA via ATRP and to determine their reactivity ratios. The reactivity ratios of styrene and EHA were calculated using Finemann–Ross (FR), inverted Finemann–Ross (IFR), and Kelen–Tudos (KT) methods (Fineman and Ross, 1950; Kelen et al., 1980; Makrikosta et al., 2005).

## Materials and methods

The monomers, 2-ethylhexyl acrylate (EHA) (Aldrich, USA) and styrene (Jyoti Chemicals, Mumbai) were purified by vacuum distillation. CuBr (Aldrich, USA) was purified by washing with glacial acetic acid, and then it was washed thoroughly with diethyl ether and was finally dried under vacuum. Phenyl ethylbromide (PEBr) (97%) and N,N,N′,N″,N″-pentamethyldiethylenetriamine (PMDETA) (97%) were purchased from Aldrich, USA and were used as received.

### Characterization

Number average molecular weight (Mn, GPC) and dispersity (***Đ***) were determined by Gel Permeation Chromatography (GPC). GPC analysis was carried out at room temperature using a Viscotek GPC equipped with a refractive index detector (Model VE3580), two ViscoGEL GPC columns (model GMHHR-M # 17392) connected in series. GPC analysis was carried out using tetrahydrofuran as eluent at a flow rate of 1 ml/min. Linear and narrow disperse polystyrene was used as calibration standard and Viscotek OMNI-01 software was used for data processing.

^1^H NMR spectra of the polymers were recorded on a 200 MHz Brucker NMR spectrometer using CDCl_3_ as solvent which had a small amount of tetramethylsilane (TMS) as an internal standard.

Differential scanning calorimetry (DSC) analysis was carried out by using TA Instrument (DSC Q100 V8.1 Build 251) under nitrogen atmosphere at a heating rate of 10°C/min within a temperature range of −100°C to +150°C. The baseline calibration was done by scanning the temperature domain with the help of an empty pan. The enthalpy was calibrated by using indium standard and the heat capacity was calibrated by using the sapphire disc that was supplied by TA instrument. The glass transition temperature (*T*_g_) was determined from the plot of heat flow vs. temperature in the second heating scan in the DSC analysis.

Thermogravimetric analysis (TGA) was carried out by using a TA Instrument (Q50) at a heating rate of 20°C/min in the temperature range of 30–600°C in nitrogen atmosphere. TGA analyzer consists of high precision balance with a pan which was placed in a small electrically heated oven. The temperature was measured accurately with the help of a thermocouple. From the plot of weight percent vs. temperature the polymer degradation temperature was determined.

### Synthesis of copolymers of styrene and EHA via atom transfer radical copolymerization (ATRcP)

The polymerization reaction was carried out in a Schlenk tube. In a typical ATRP reaction EHA (4.82 g, 26.1 mmol), styrene (0.909 g, 8.7 mmol) and CuBr (0.050 g, 0.35 mmol) were accurately weighed and transferred to the Schlenk tube. The PMDETA ligand (0.0604 g, 0.35 mmol) was then added to the reaction tube. Oxygen was removed from the reaction mixture by passing nitrogen through the reaction tube. The polymerization was started by adding PEBr (0.0646 g, 0.35 mmol) and was carried out at 90°C. Aliquot samples were taken out at different time intervals and were used to calculate the conversion by gravimetric method. The samples were also used to find out the molecular weight by GPC. The final product was diluted with THF and was purified by passing through alumina column to remove the copper catalyst. The same procedure was adopted for other feed ratios of ATRcP (atom transfer radical copolymerization) of EHA and styrene.

### Homopolymerization of styrene via atom transfer radical polymerization (ATRP)

The homopolymerization of styrene was carried out in bulk in a Schlenk tube equipped with silicone septum and magnetic stirring bar. In the Schlenk tube styrene (4.5 g, 43.2 mmol), CuBr (0.031 g, 0.21 mmol) and PMDETA (0.037 g, 0.21 mmol) were weighed and degassed by passing nitrogen gas for 15 min. The reaction was started by adding PEBr (0.040 g, 0.21 mmol) in the mixture. The reaction was carried out at 110°C for 6 h. ^1^H NMR (CDCl_3_, 200 MHz): δ (in ppm) = 6.4–7.2 (phenyl protons of polystyrene) and 1.4–2.2 ppm (−CH_2_− and >CH− protons).

### Synthesis of diblock copolymer of polystyrene with EHA via ATRP

The homopolymer of polystyrene (i.e., PS-Br) was used as macroinitiator for the synthesis of diblock copolymer of styrene and EHA. The macroinitiator, PS-Br (1.0 g, 0.066 mmol) was taken in a Schlenk tube and was dissolved in THF solvent into which CuBr (0.009 g, 0.069 mmol) and PMDETA (0.017 g, 0.10 mmol) were added followed by EHA (1 g, 5.4 mmol). The polymerization reaction was carried out at 90°C for 6 h. The polymer obtained was dissolved in THF and was purified by passing through basic alumina column and then precipitated in methanol.

## Results and discussion

**Scheme 1:**
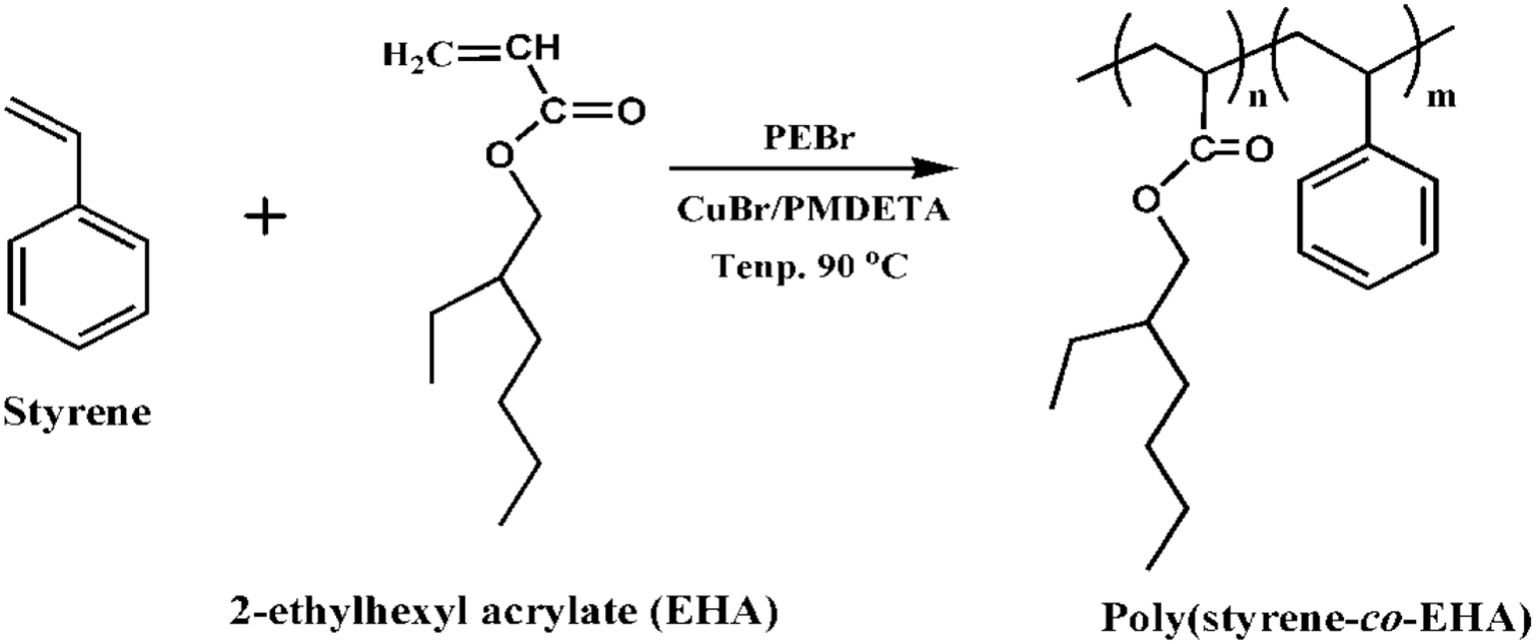
**Copolymerization of styrene and EHA via atom transfer radical polymerization (ATRP)**.

**Figure 1:**
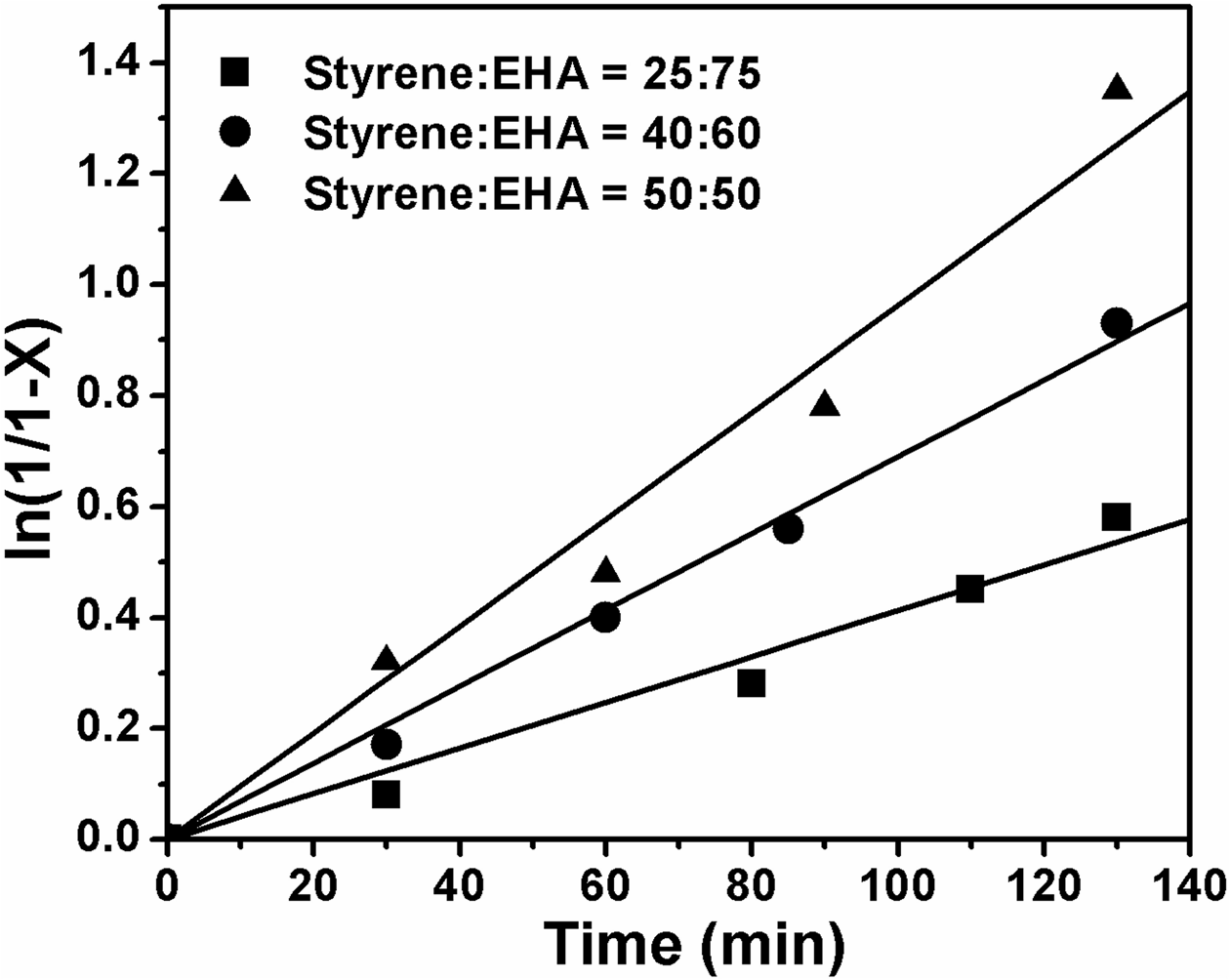
**Kinetic plot of ln[1/(1-X)] vs time for copolymerization of styrene and EHA**. [PEBr]: [M]_o_:[CuBr]:[PMDETA] = 1:100:1:1, at 90°C.

**Figure 2:**
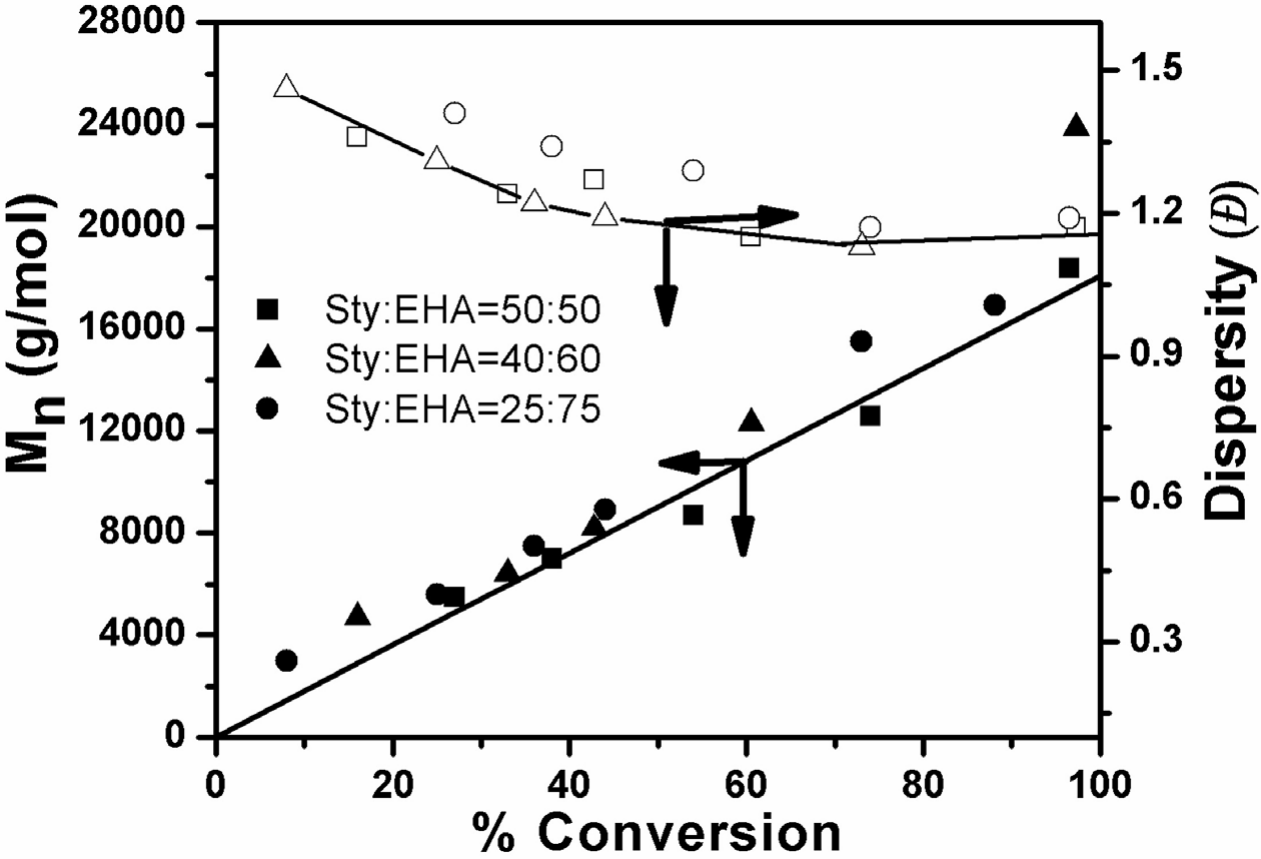
**Plot of *M*_n_ and ***Đ*** vs. conversion (%) for ATRcP of Styrene and EHA in bulk**. [PEBr]: [M]_o_:[CuBr]:[PMDETA] = 1:100:1:1, at 90°C.

Copolymerization of styrene and 2-ethylhexyl acrylate was carried out via ATRcP (shown in Scheme 1) at different feed ratios by using phenylethylbromide (PEBr) as initiator, CuBr as catalyst in combination with PMDETA as a ligand. From the kinetic plot of ln(1/1-X) (where X is the percent conversion of monomer) vs. time, it was observed that the value of ln(1/1-X) was linearly increased with time (Figure 1). This linear dependency is the characteristics of the controlled polymerization reaction which follows the first order kinetics. Figure 1 showed that with increase in styrene content in the feed the rate of polymerization increased (Jianying et al., 2006). Figure 2 infers that there was linear increase in molecular weight with conversion and dispersity (***Đ***) was relatively narrow. GPC traces of poly(styrene-co-EHA) (50:50) (sample 3 of Table 1) is shown in supplementary section (Figure S1). It indicates the controlled nature of the copolymerization reaction. Table 1 summarizes the feed ratio as well as the copolymer composition of the different copolymerization reactions.

**Table 1:** **Copolymerization of styrene and EHA in bulk at 90°C**.

**Sl. No**.	**Feed composition (%) styrene: EHA**	**Copolymer composition^≠^ (%) styrene: EHA**	**Conversion (%)**	***M*_*n*_,GPC(g/mol)**	***Đ***
1	25:75	27:73	98.1	16,900	1.27
2	40:60	44:56	88.0	24,000	1.15
3	50:50	56.6:43.4	97.8	18,500	1.17

*[PEBr]: [M]_o_:[CuBr]:[PMDETA] = 1:100:1:1. Reaction time = 2 h 20 min*.

≠ *Compositions were calculated by ^1^H NMR spectroscopy*.

### Structural characterization and copolymer composition

**Figure 3:**
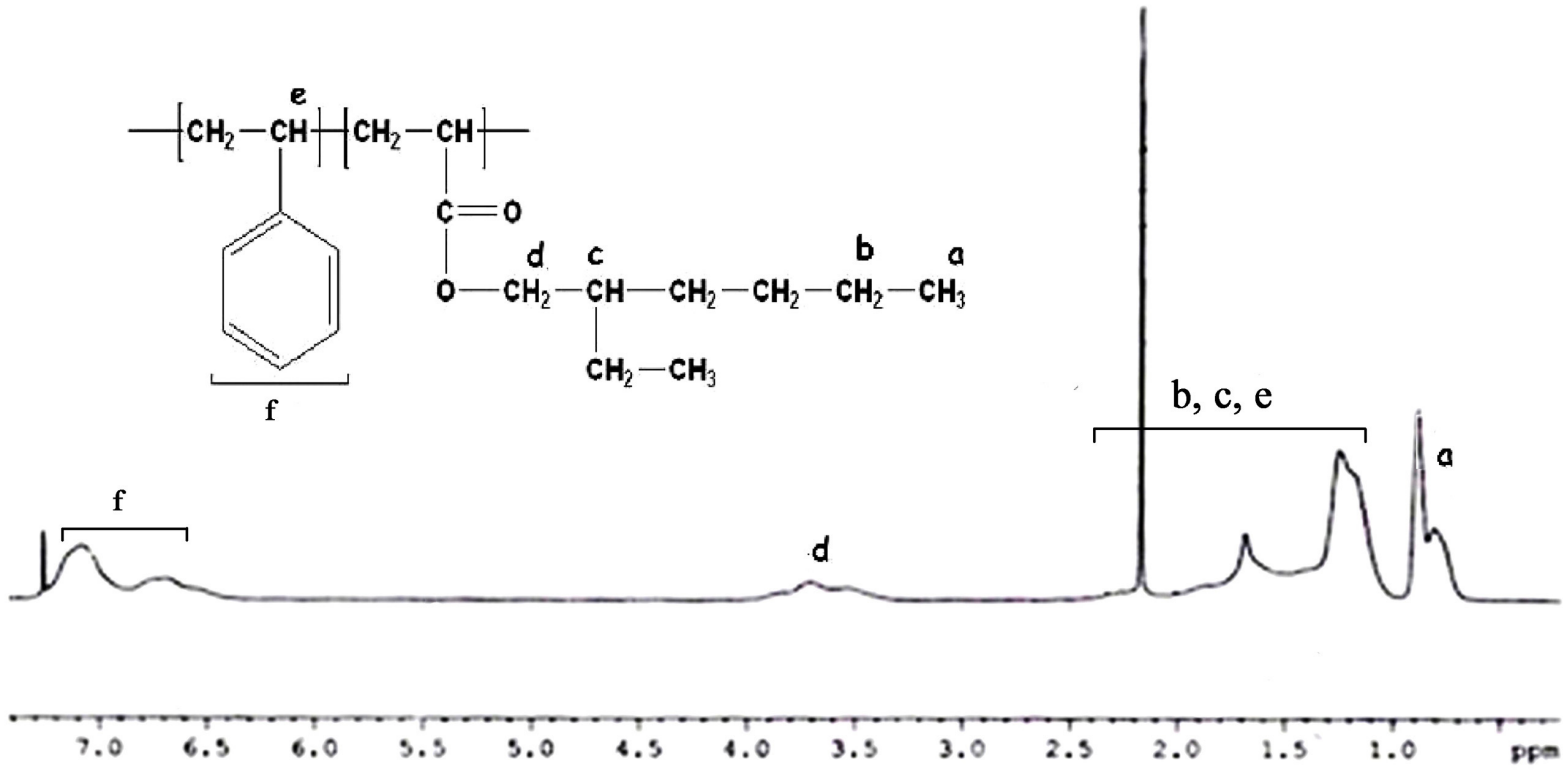
**^1^H NMR spectrum of poly(styrene-*co*-EHA) with feed molar composition 50:50**.

The structural characterization and copolymer composition were determined by ^1^H NMR spectroscopy. Figure 3 shows the ^1^H NMR spectra of poly(styrene-*co*-EHA) of 40:60 feed ratio. The resonance at δ = 0.8 ppm is due to the –CH_3_ protons of PEHA part. The broad resonances at δ = 1.0 to 2.1 ppm are due to the different –CH_2_– and >CH– protons of pendant group of PEHA part as well as those of the main chain backbone of the copolymer. The resonances at δ = 6.5–7.1 ppm are due to the different aromatic protons of polystyrene part. The resonances at 3.8 ppm are due to –OCH_2_– protons of PEHA part. The distinct resonances at 6.5–7.3 for five aromatic protons of styrene and 3.8 ppm for two protons of EHA were used to calculate the composition of the copolymer of styrene and EHA. The copolymer composition was determined by the equation (1) as shown below

(1) $$F_{EHA}=\frac{5A}{5A+2B}\times 100$$

where, A and B represent the integral area at δ = 3.7 and δ = 6.5–7.3 ppm for –OCH_2_– protons in PEHA and aromatic protons of polystyrene unit respectively.

### Reactivity ratio determination

In the copolymerization of styrene (1) and EHA (2) the reactivity ratio r_1_ is defined as k_11_/k_12_, where k_11_ is the rate constant of the reaction between the growing polymer chain carrying free radical of styrene as the terminal unit and styrene (homo propagation) and k_12_ is the rate constant of the reaction between the same reactive chain end and the EHA monomer. Similarly the reactivity ratio r_2_ is also defined as k_22_/k_21_, where k_22_ is the rate constant of homo propagation reaction between the growing macromolecular chain having EHA active radical as the terminal unit and EHA and k_21_ is the rate constant of the reaction between EHA active radical and styrene monomer (cross propagation). For determining the r_1_ and r_2_, copolymerization of styrene and EHA was carried out at different feed ratio of styrene (1) and EHA (2). In this case copolymerization was carried out at low conversion (~10%) and its molar composition was determined by ^1^H NMR spectroscopy. Composition of the low conversion copolymer was used for the determination of monomer reactivity ratios. In this case FR, IFR, and KT methods were used to determine the reactivity ratio of the monomers.

In the FR method the following equation was used

$$\text{M}-\frac{\text{M}}{\text{P}}=-{\text{r}}_{2}+{\text{r}}_{1}\frac{{\text{M}}^{2}}{\text{P}}$$

where, M = molar feed ratio (M_1_/M_2_) and P = copolymer composition (m_1_/m_2_)

$$\text{or},\text{ G}=-{\text{r}}_{2}+{\text{r}}_{1}\text{H}$$

where, G = M−M/P and H = M^2^/P

**Figure 4:**
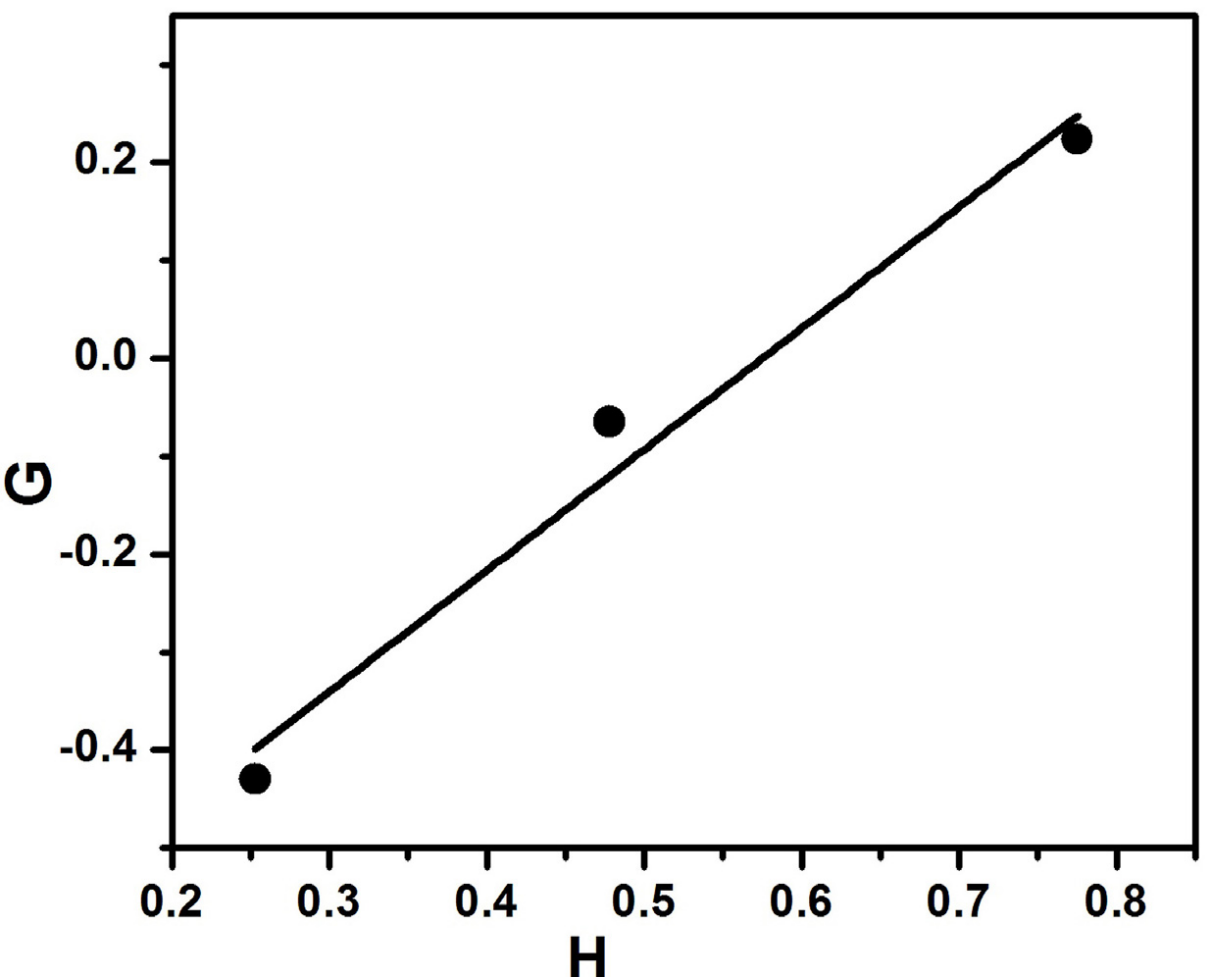
**Finemann–Ross plot for copolymerization of styrene with EHA**.

The plot of G vs. H gives the straight line (Figure 4). From this the slope and intercept were calculated to be r_1_ = 1.24 and r_2_ = 0.71 respectively.

In the IFR method the equation used is

$$\frac{\text{G}}{\text{H}}=-{\text{r}}_{2}\frac{1}{\text{H}}+{\text{r}}_{1}$$

**Figure 5:**
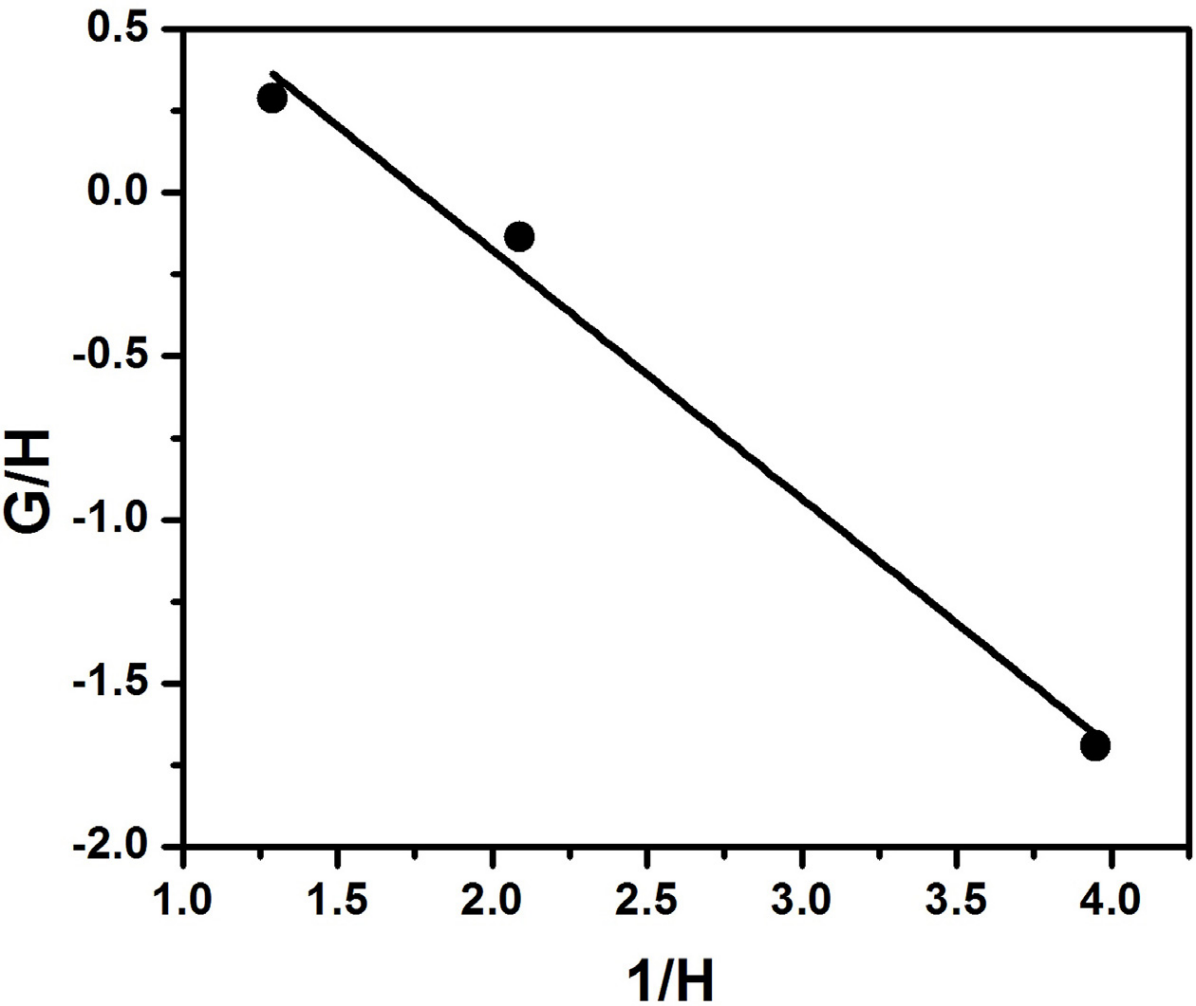
**Inverted Finemann–Ross plot for copolymerization of styrene with EHA**.

So, from the plot of G/H and 1/H (Figure 5) the reactivity ratios, r_1_ and r_2_ were calculated as 1.34 and 0.76 from the intercept and the slope respectively.

In KT method the reactivity ratio was determined by using the equation

$$\eta =\left({\text{r}}_{1}+\frac{{\text{r}}_{2}}{\propto }\right)\xi -\frac{{\text{r}}_{2}}{\propto }$$

where, η = G/(∝+H) and ξ = H/(∝+H) and ∝ = (H_min_. H_max_)^1/2^, H_min_ = 0.253 and H_max_ = 0.775. H_min_ and H_max_ values are taken from Table 2.

**Table 2:** Finemann–Ross, Inverted Finemann–Ross and Kelen–Tudos parameter for the copolymer of styrene and EHA in bulk^*^.

**Monomer feed ratio styrene: EHA**	**M = M_1_/_2_**	**^#^*P* = m_1_/_2_**	***M*/*P***	**P-1**	***G***	***H***	***G*/*H***	**1/H**	**∝+H**	**η**	**ξ**
25:75	0.33	0.43	0.767	−0.57	−0.430	0.253	−1.69	3.95	0.695	−0.618	0.364
40:60	0.66	0.91	0.725	−0.09	−0.065	0.478	−0.135	2.09	0.920	−0.070	0.519
50:50	1.0	1.29	0.775	0.29	0.224	0.775	0.289	1.29	1.217	0.184	0.636

* *[PEBr]: [M]_o_:[CuBr]:[PMDETA] = 1:100:1:1, at 90°C (where, ∝ = 0.442)*.

# *Copolymer composition at 10% conversion*.

**Figure 6:**
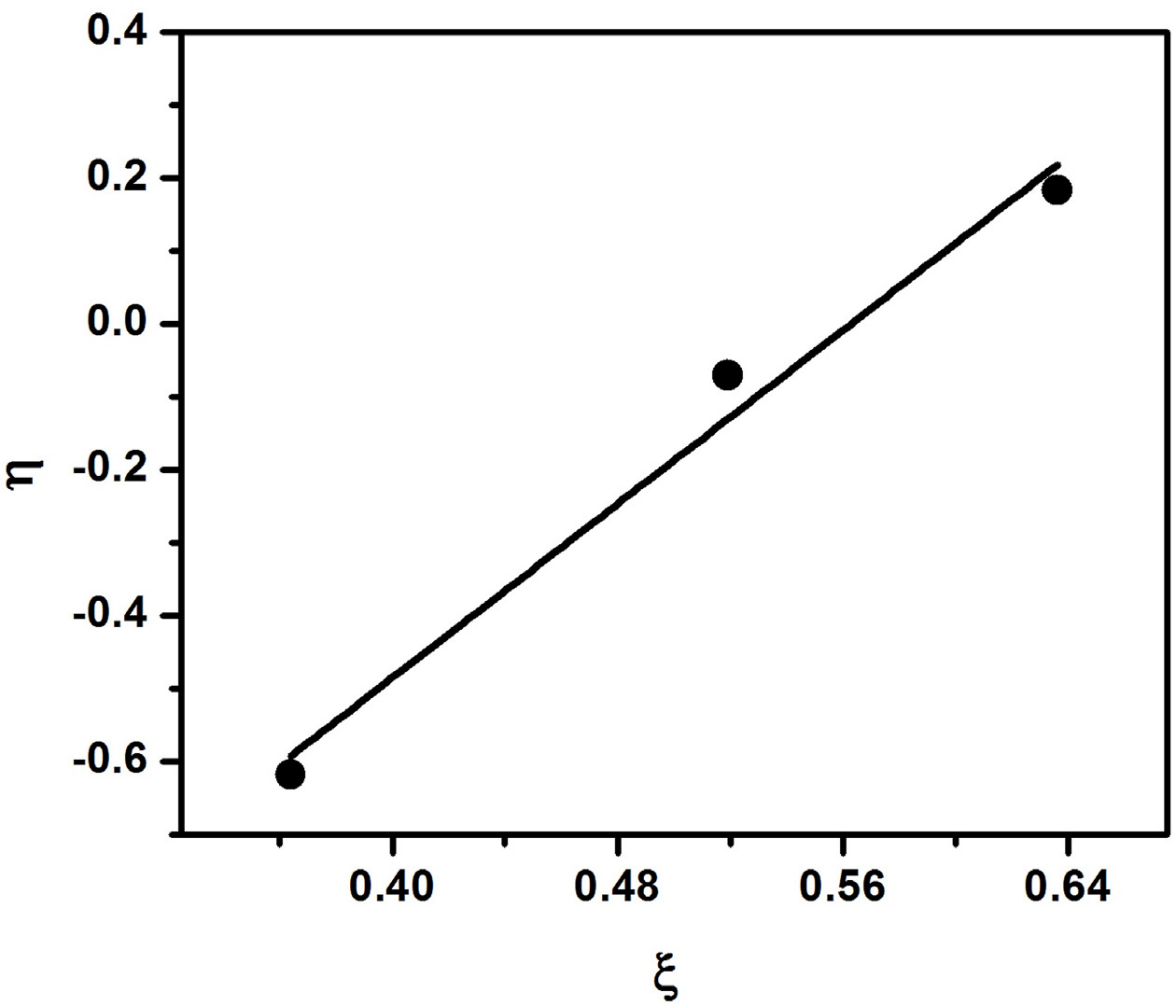
**Kelen-Tudos plot for copolymerization of styrene with EHA**.

In the plot of η vs. ξ, (Figure 6) the slope gives the value of (r_1_ + r_2_/∝) and intercept provides r_2_/∝. From these two values r_1_ and r_2_ were calculated as r_1_ = 1.30 and r_2_ = 0.73 respectively. All the parameters used for the three methods are given in Table 2.

The reactivity ratios, r_1_ and r_2_ calculated by the different methods are tabulated in Table 3. They are quite comparable. The values; r_1_ > 1, r_2_ < 1 and r_2_ < r_1_ indicate that the styrene has much influence on the copolymer formation during the reaction (Jianying et al., 2006). Srivastava et al. (Srivastava and Rai, 1995) reported the copolymerization of styrene and EHA initiated by azobisisobutyronitrile (AIBN) in bulk in the presence of anhydrous ZnCl_2_. They reported the reactivity ratios for styrene and EHA as 0.10 and 0.175 respectively. Moreover, Kavousian et al. reported the copolymerization of styrene and EHA via conventional radical polymerization (Kavousian et al., 2004). They reported the reactivity ratio of styrene and EHA as 0.926 and 0.238 respectively. There is a difference in the polymerization mechanism of ATRP and non-ATRP processes. In ATRP, atom transfer from an organic halide to a transition metal complex occurs to generate the active radical species, which are then quickly “deactivated” by back transfer of the atom from the transition metal to the radical species (Matyjaszewski, 2002; Braunecker and Matyjaszewski, 2007). So, there is difference in reactivity ratio values in comparison to FRP. Barim et al. studied the FRP and ATRP of phenoxycarbonylmethyl methacrylate (PCMMA) and styrene at 110°C. They reported the reactivity ratios of PCMMA and styrene prepared via ATRP were 0.33 and 0.96 respectively and the same prepared via FRP were 0.47 and 1.16 respectively (Barim et al., 2007). The reactivity ratio varies with the polymerization temperature (Chambard et al., 1999; McManus et al., 2002). We did the polymerization reaction via ATRP in bulk at 90°C. However, the reactivity ratios calculated in this work follow the same trend (r_EHA_ < r_styrene_) as reported by the other authors. In addition, in ATRcP technique the product of the reactivity ratios is less than one. It shows the tendency of random copolymer formation, where the chances of incorporation of styrene is more in comparison to EHA.

**Table 3:** **Reactivity ratio of styrene (r_1_) and EHA (r_2_) determined by three different models**.

**Methods used**	**r_1_ (styrene)**	**r_2_ (EHA)**	**r_1_.r_2_**
FR	1.24	0.71	0.88
IFR	1.34	0.76	1.01
KT	1.30	0.73	0.91

The mean sequence length (l) of the copolymers were determined by using the equations (Pazhanisamy et al., 1997)

$$l_{1}={\text{r}}_{1}\frac{{\text{M}}_{1}}{{\text{M}}_{2}}+1$$

and

$$l_{2}={\text{r}}_{2}\frac{{\text{M}}_{2}}{{\text{M}}_{1}}+1$$

where, r_1_ (styrene) = 1.29 and r_2_ (EHA) = 0.73

The results of the mean sequence length in the copolymer are shown in Table 4. It indicates that the length of EHA increases as its content in the monomer feed increases.

**Table 4:** **The monomer composition and sequence length ratio**.

**Copolymer feed ratio styrene: EHA**	**M_1_/M_2_**	**M_2_/M_1_**	**l_1_**	**l_2_**	**l_1_: l_2_**
25/75	0.33	3.00	1.42	3.19	4:9
40/60	0.66	1.50	1.85	2.09	4:5
50/50	1.00	1.00	2.29	1.73	4:3

### Thermal properties

**Figure 7:**
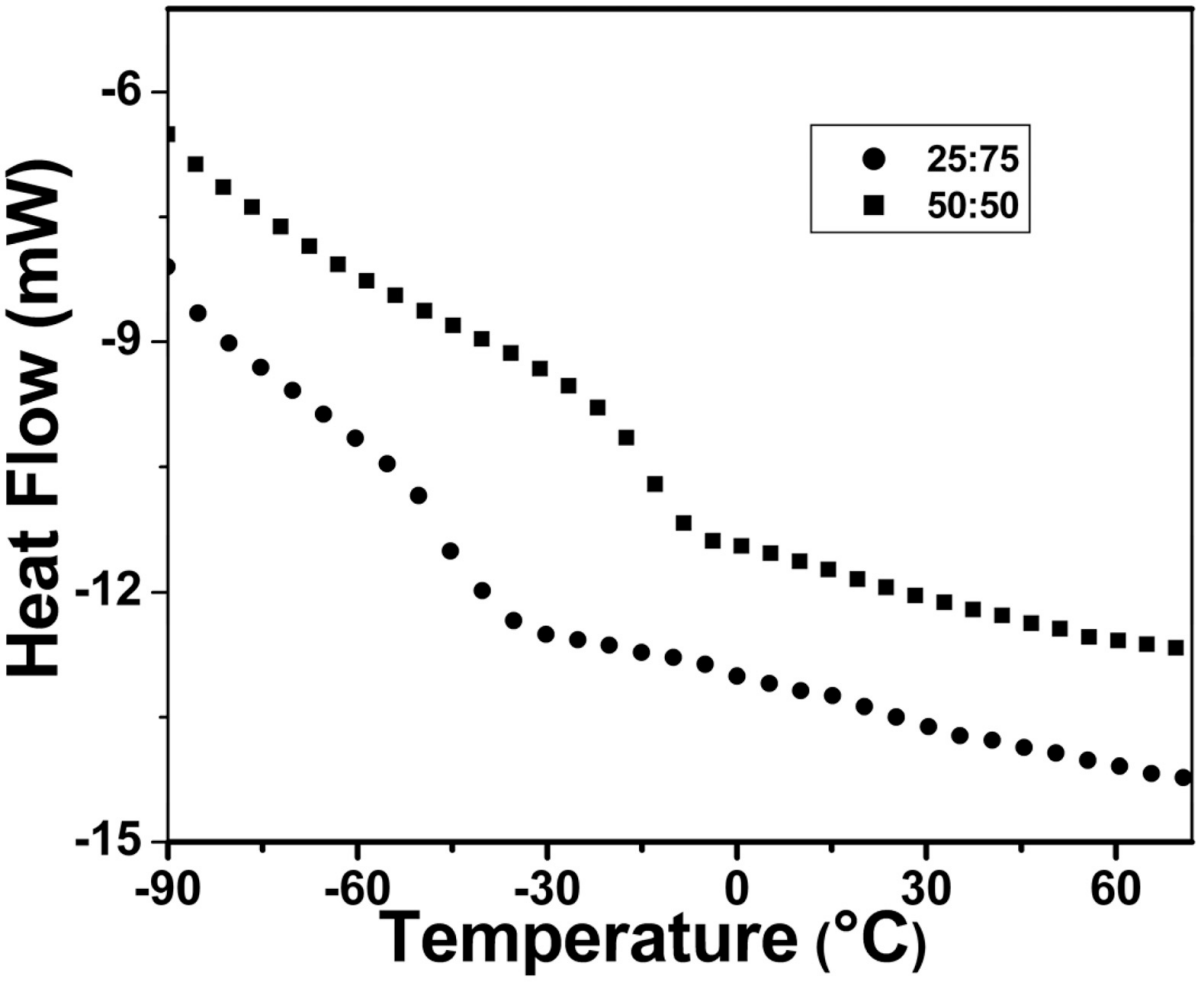
**DSC thermogram of poly(styrene-*co*-EHA)**.

The glass transition temperature (*T*_g_) of the copolymer was determined by DSC analysis (shown in Figure 7). All the copolymers showed a single *T*_g_ and they are shown in Table 5. It indicates that as the EHA (*T*_g_ = −60^°^C) content increases the *T*_g_ decreases. The *T*_g_ for the copolymers determined by DSC analysis was compared with the same (*T*_g, F-F_) determined by Flory-Fox equation (Lijia et al., 1997). There is consistent difference between the *T*_g_ values at different content of two comonomers (Table 5). Table 5 shows that there is some discrepancy between the experimental *T*_g_ and *T*_g_ calculated by Flory-Fox equation. The Flory-Fox model is based on the free volume theory. The discrepancy in the *T*_g_ values is due to the fact that the effect of the chemical nature and organization of the monomers on the mobility of a polymer chain was not considered (Fernandez-Garcia et al., 1999).

**Table 5:** **Thermal properties of copolymers of styrene and EHA**.

**Sl no**.	**Feed composition styrene: EHA**	**Copolymer composition styrene: EHA**	***T*_g_(^°^C)**	^*^T_g,F-F_(^°^C)	**^#^*T*_onset_(^°^C)**	***T*_max_(^°^C)**
1	25:75	27:73	−47	−36	381	409
2	40:60	44:56	−27	−18	389	414
3	50:50	56.6:43.4	−14	−5	385	415
4	Polystyrene-b-PEHA	85: 15	+123 and −65	–	–	–

* *Tg,F-F was calculated by using Flory–Fox equation*.

# *Temperature at 10% weight loss was taken as T_onset_*.

*T_max_ represents the maximum degradation temperature at which the polymer back-bone starts degrading. It was determined from the DTG thermogram*.

**Figure 8:**
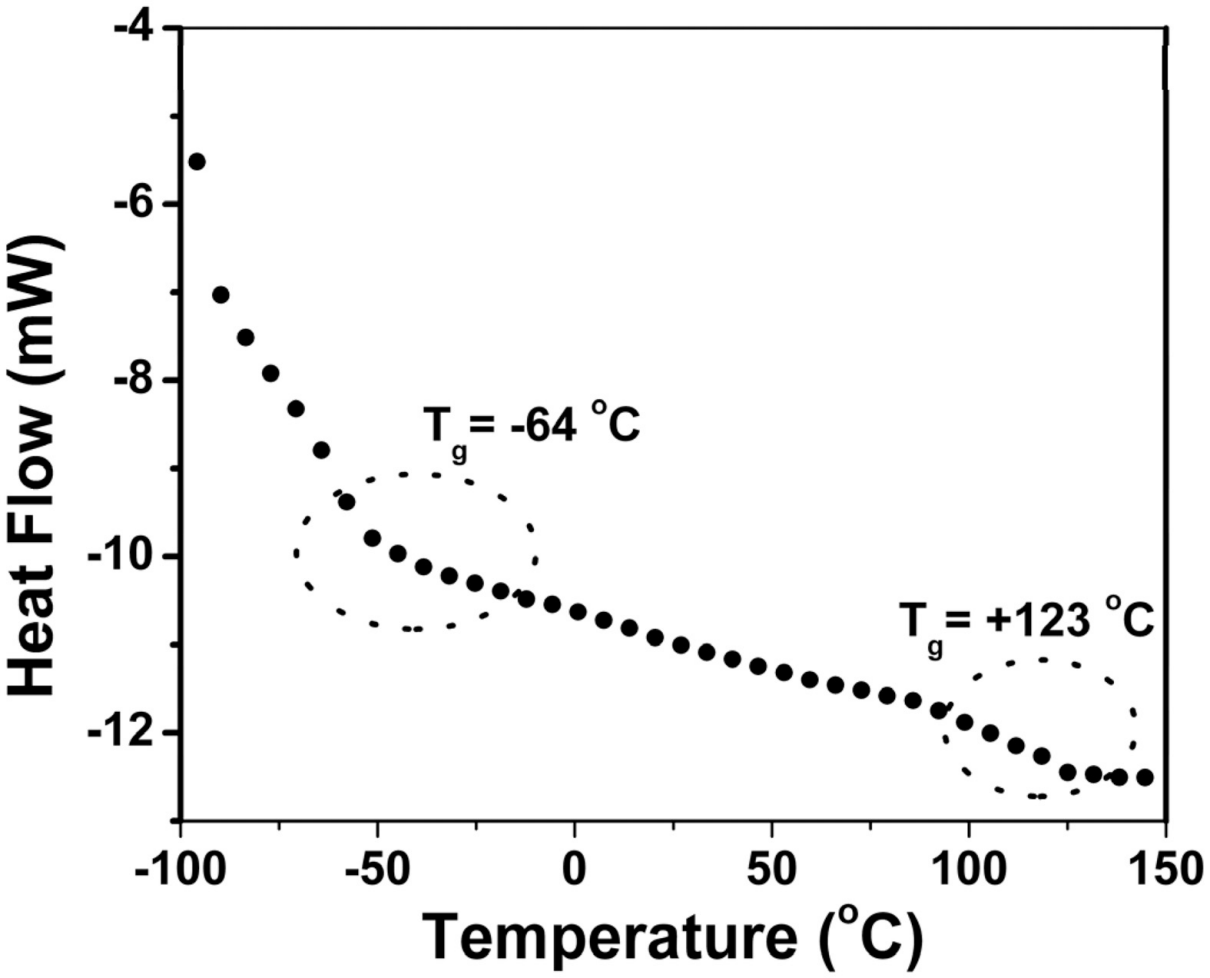
**DSC thermogram of the polystyrene-*b*-PEHA**.

**Figure 9:**
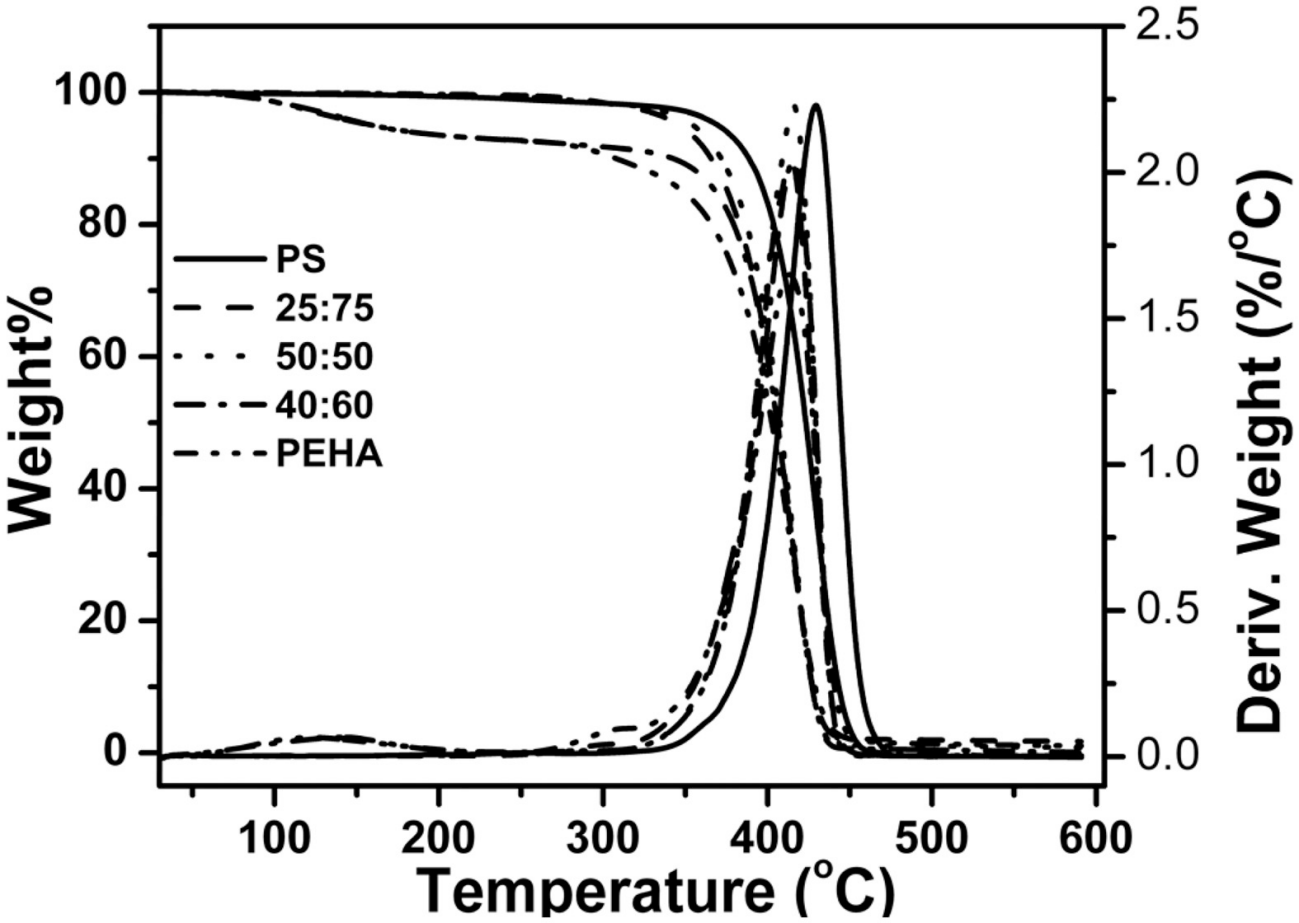
**TGA thermogram of homopolymer and copolymers of styrene and EHA**.

The block copolymer of styrene and EHA (PS-*b*-PEHA) was prepared by using polystyrene-Br as macroinitiator. The shift of GPC traces of the diblock copolymer toward lower elution volume indicated the successful preparation of block copolymer. (The GPC traces are shown in Figure S2). This block copolymer showed two *T*_g_s, PEHA block at −64°C and polystyrene at +123°C (Figure 8). However, the copolymers, poly(styrene-*co*-EHA) showed only one *T*_g_ indicating the copolymers were not blocky in nature. Thermal stability of the copolymer was studied by TGA (Figure 9). It is clear that as the styrene content increases, there is a slight increase in *T*_onset_. However, there was no significant change in *T*_max_ as shown in Table 5.

## Conclusions

Copolymers of styrene and 2-ethylhexyl acrylate were synthesized successfully in bulk by using atom transfer radical copolymerization (ATRcP). The chemical composition was studied by ^1^H NMR spectroscopy and the reactivity ratios of the two monomers were calculated by using FR, IFR, and KT methods. The reactivity ratios of styrene and EHA were somewhat different from the polymerization reaction of styrene and EHA using the FRP system. DSC analysis showed that the *T*_g_ of the copolymer increases on increasing styrene content.

### Conflict of interest statement

The authors declare that the research was conducted in the absence of any commercial or financial relationships that could be construed as a potential conflict of interest.

## Supplementary material

Figure S1 **GPC traces of poly(styrene-*co*-EHA), sample 3 of Table 1**.

Figure S2 **GPC traces of polystyrene macroinitiator and polystyrene-*b*-PEHA block copolymer**.
