# Supplementary figures and images for: Copper mediated controlled radical copolymerization of styrene and 2-ethylhexyl acrylate and determination of their reactivity ratios
Source: Front Chem. 2014 Oct 17;2:91. doi: 10.3389/fchem.2014.00091 (PMC4201150; doi:10.3389/fchem.2014.00091)

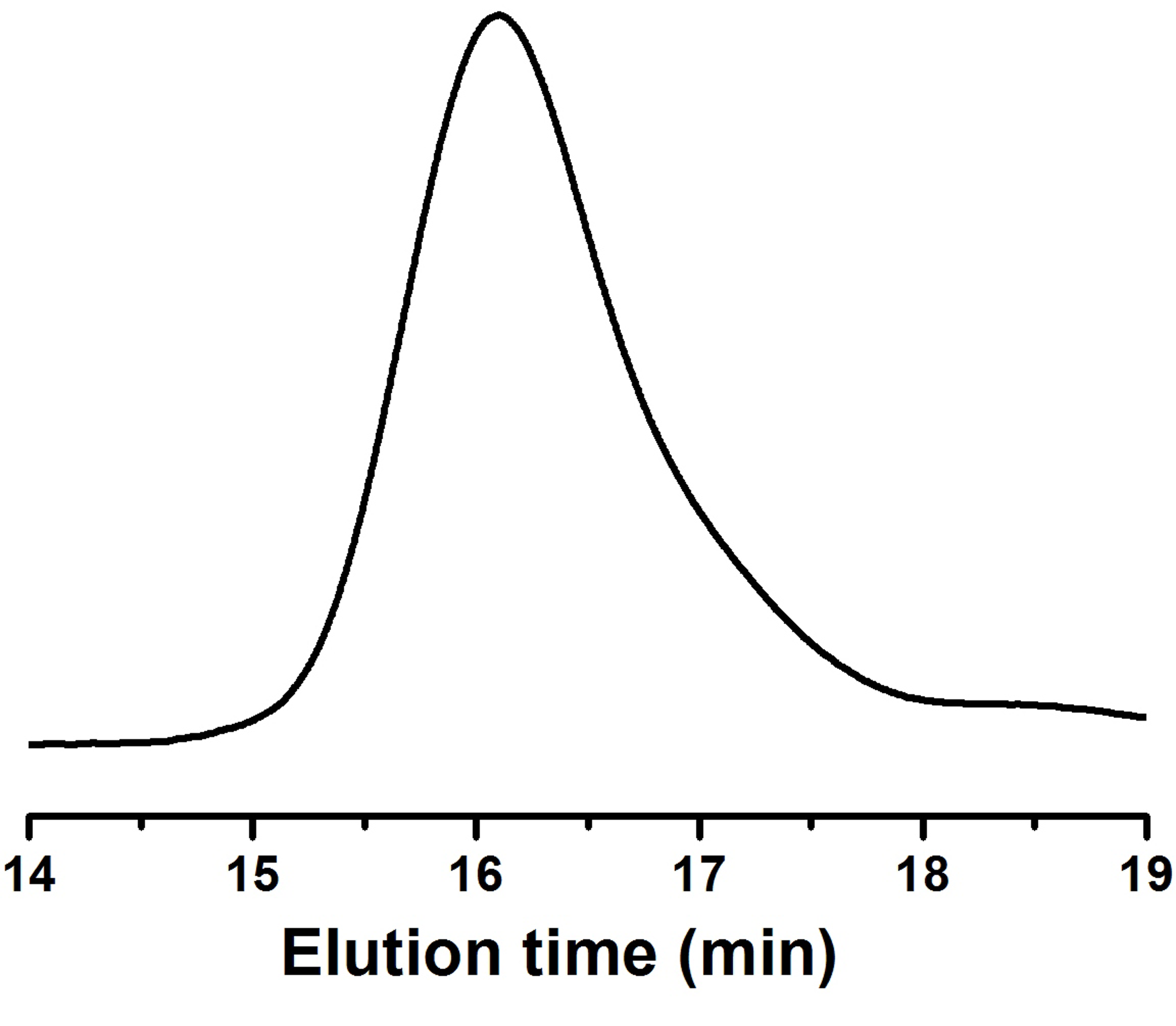

Supplement: Figure S1 — GPC traces of poly(styrene-co-EHA), sample 3 of Table 1. [file Image1.TIF]

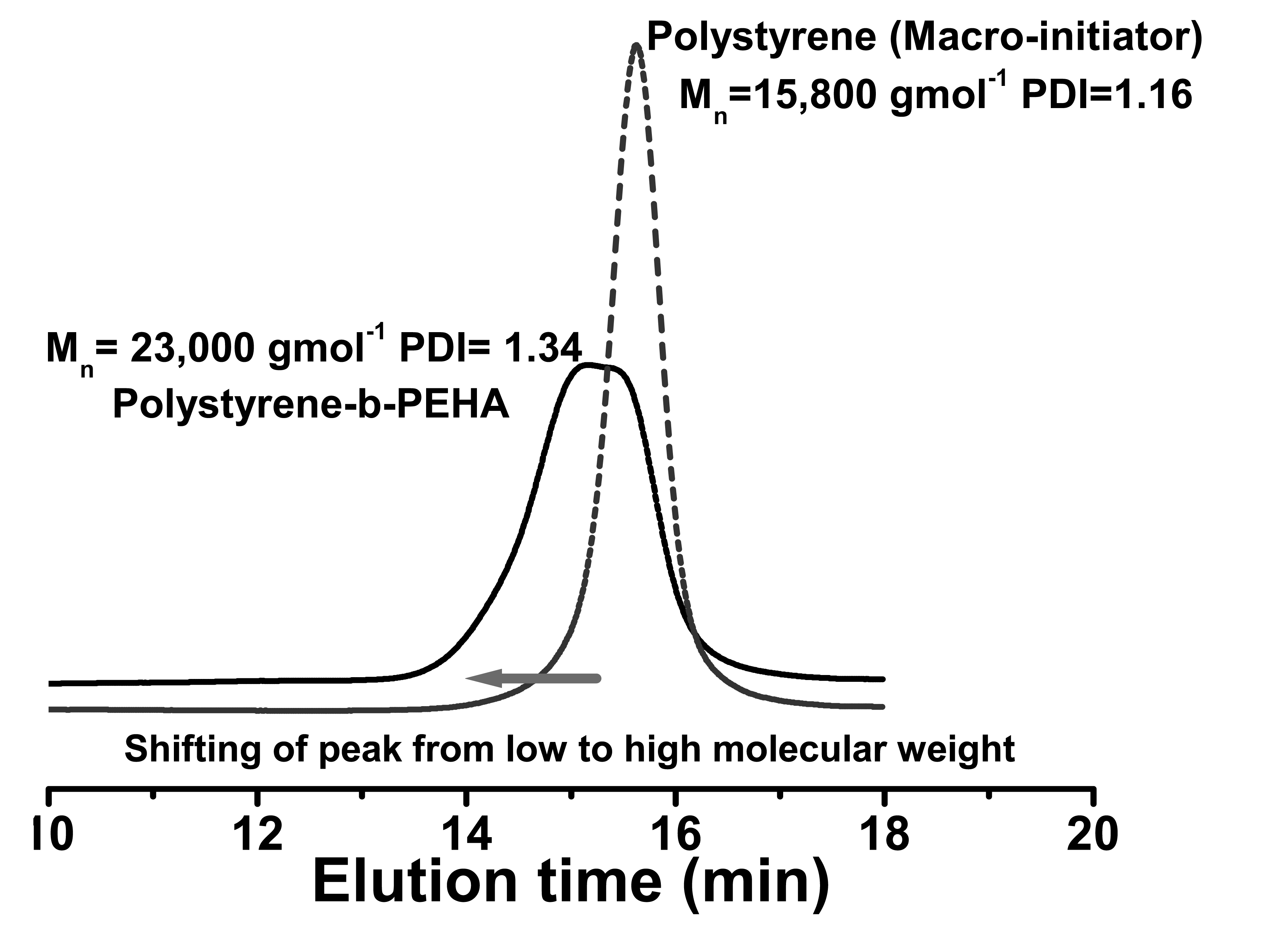

Supplement: Figure S2 — GPC traces of polystyrene macroinitiator and polystyrene-b-PEHA block copolymer. [file Image2.TIF]
